# Soy peptide as an antidote to undernutrition

**DOI:** 10.1093/lifemeta/loae034

**Published:** 2024-08-21

**Authors:** Mark P Mattson

**Affiliations:** Department of Neuroscience, Johns Hopkins University, Baltimore, MD 21205, USA

**The recent study by Xu *et al*., published in *Advanced Science*, offers a groundbreaking approach to addressing undernutrition using soy peptide supplementation in a novel non-human primate (NHP) model. This research provides valuable insight into the metabolic mechanisms underlying undernutrition and introduces a promising therapeutic strategy.**

**Figure 1:**
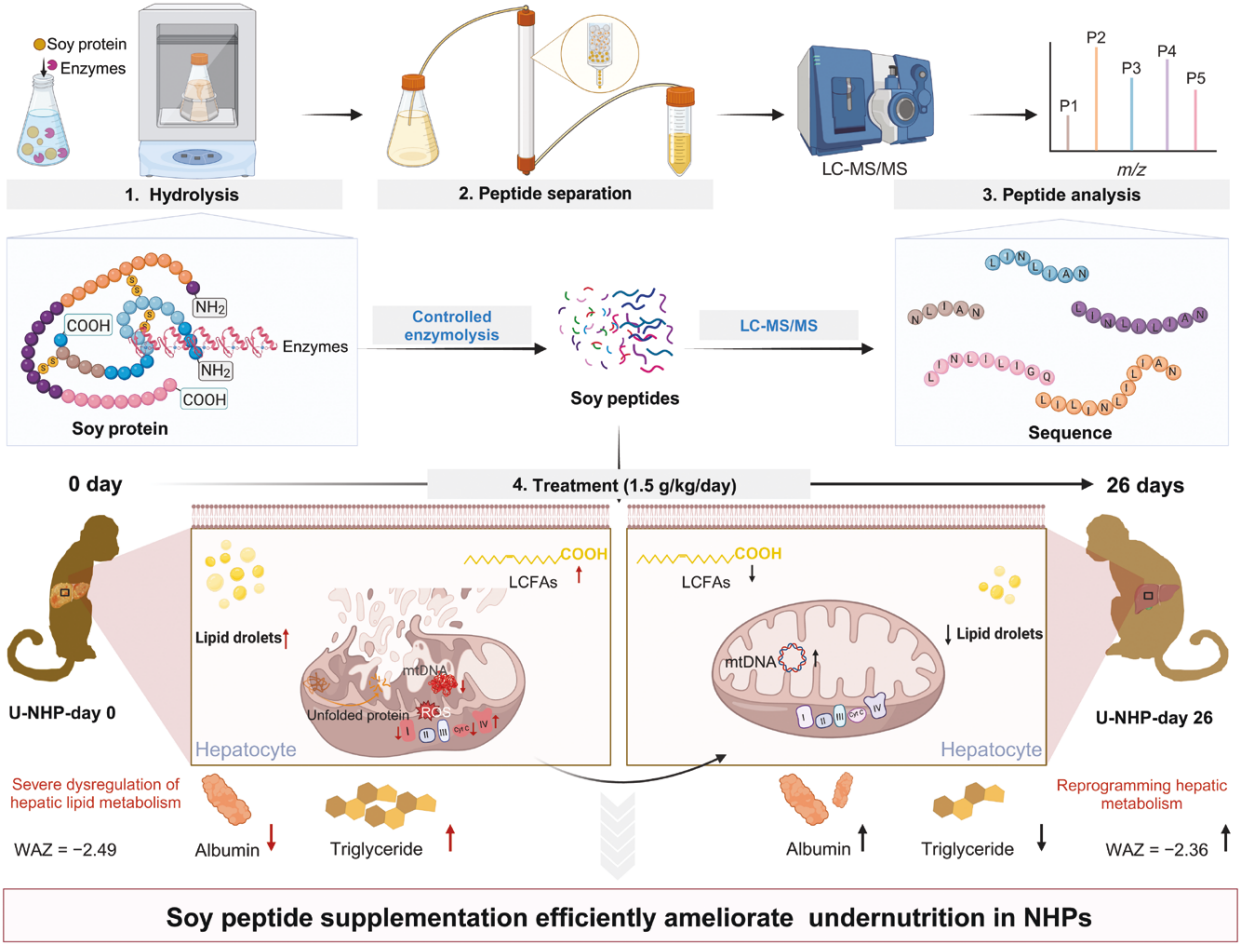
Experimental design and key findings of the study. Soy protein was subjected to controlled enzymatic hydrolysis to produce soy peptides which were then analyzed using liquid chromatography–mass spectrometry (LC–MS/MS). NHPs were treated with soy peptides for 26 days. Biochemical analyses included measurements of albumin and triglyceride levels on day 0 and day 26, and monitoring of long-chain fatty acids (LCFAs), lipid droplets, and reactive oxygen species (ROS) in hepatocytes. The impact on mitochondrial electron transport chain complexes (I–IV), cytochrome C (Cyt C), and mitochondrial DNA (mtDNA) was also assessed, along with the status of unfolded proteins and hepatic lipid metabolism. The results showed that soy peptide supplementation significantly ameliorated undernutrition in NHPs, as indicated by an improvement in WAZ scores from −2.49 to −2.36. Additionally, there was severe dysregulation of hepatic lipid metabolism in the untreated state, which was reprogrammed towards a healthy state following soy peptide treatment.

Using a large cohort of 1636 non-human primates (NHPs; *Macaca fascicularis*), the researchers established a weight-for-age z-score (WAZ) of ≤ −1.83 as the criterion for identifying undernourished animals [1] (Fig. 1). This model closely mimics human undernutrition, overcoming the limitations of previous rodent and piglet models [2]. The study revealed severe hepatic metabolic dysregulation as a hallmark of undernutrition, specifically implicating mitochondrial dysfunction rather than peroxisome impairment. To investigate the therapeutic potential of soy peptides, the authors derived these peptides from soy protein using a controlled enzymatic hydrolysis technique. They administered the soy peptides and observed significant improvements in WAZ scores and reversal of pathological anomalies. The soy peptides effectively reprogrammed hepatic lipid metabolism, restored mitochondrial function, and mitigated hepatic steatosis. Biochemical parameters, including serum albumin, total proteins, triglycerides, and inflammatory markers, were also significantly improved. Detailed biochemical and histological analyses confirmed the systemic benefits of soy peptide supplementation. The researchers observed amelioration of liver, spleen, kidney, and muscle pathologies in response to soy peptides.

The innovative use of a NHP model in this study is a significant advancement. Because of NHPs’ close genetic, physiological, and metabolic similarities to humans, the findings of Xu *et al*. provide a more accurate representation of human disease states compared to traditional rodent models. The model used in this study successfully replicated the complex pathophysiology of human undernutrition, providing a robust platform for exploring therapeutic interventions. One of the key findings of this study was the identification of mitochondrial dysfunction as a central feature of undernutrition-induced hepatic steatosis. The study revealed that undernourished NHPs exhibited severe mitochondrial abnormalities, including reduced mitochondrial number, disrupted oxidative phosphorylation (OXPHOS) complexes, and impaired fatty acid oxidation. These mitochondrial abnormalities were identified as critical drivers of hepatic lipid accumulation and overall metabolic dysregulation, which is different from previous studies of rodents [3].

The World Health Organization (WHO) has long emphasized the importance of addressing undernutrition, which affects millions of children and adults globally [4]. Undernutrition contributes significantly to morbidity and mortality rates, especially in developing countries. WHO’s strategies include promoting breastfeeding, improving dietary diversity, and providing micronutrient supplements [5]. However, the persistent challenge is finding sustainable and effective treatments that can address the root causes of undernutrition rather than just its symptoms. As demonstrated in this study, soy peptides offer a promising solution. By reprogramming hepatic metabolism and improving mitochondrial function, soy peptides address the core metabolic dysfunctions associated with undernutrition. This mechanism-based approach aligns with WHO’s goals of providing sustainable and effective nutritional interventions. Moreover, the safety and natural origin of soy peptides make them an attractive option for widespread use in vulnerable populations [6].

The study’s implications extend beyond clinical applications to agricultural and food production sectors. The ability to produce soy peptides through enzymatic hydrolysis of soy proteins means that this therapeutic intervention can be scaled up and integrated into existing food production systems. This scalability is particularly relevant for regions with high undernutrition rates, such as parts of Africa and Southeast Asia, where local production of soy-based supplements could provide a cost-effective and sustainable solution. By applying enzymatic hydrolysis to different protein-rich crops, it is possible to develop a range of peptide supplements tailored to regional dietary practices and available resources [7]. This flexibility in production could enhance the sustainability and cultural acceptability of nutritional interventions.

While the study by Xu *et al*. offers promising insights and potential solutions for managing undernutrition in humans, additional research is required for translation to humans. The study’s focus on one specific cohort of *Macaca fascicularis* limits the generalizability of the findings to other primate species and potentially to humans. The long-term effects and safety of soy peptide supplementation were not evaluated. Moreover, the scalability and economic feasibility of producing and distributing soy peptides on a global scale, particularly in resource-limited settings, remain challenges that would need to be addressed [8]. Future research should aim to validate these findings in human clinical trials, explore the long-term impacts of supplementation, and assess the logistical aspects of large-scale production and distribution.

In summary, the study by Xu *et al*. provides robust evidence for the use of soy peptides as a therapeutic intervention for undernutrition. By reprogramming hepatic metabolism and addressing mitochondrial dysfunction, soy peptides offer a novel and effective approach to managing undernutrition. This research represents a significant advancement in nutritional science, with the potential to transform the treatment of undernutrition and improve the health and well-being of affected populations worldwide.
