# Correction to: Soy peptide as an antidote to undernutrition

**DOI:** 10.1093/lifemeta/loaf007

**Published:** 2025-03-10

This is a correction to: Mark P Mattson, Soy peptide as an antidote to undernutrition, *Life Metabolism*, Volume 3, Issue 5, October 2024, https://doi.org/10.1093/lifemeta/loae034

The article title has been updated to address a typographical error: “anecdote” now reads “antidote”.

